# A Lentivirus-Mediated Genetic Screen Identifies Dihydrofolate Reductase (DHFR) as a Modulator of β-Catenin/GSK3 Signaling

**DOI:** 10.1371/journal.pone.0006892

**Published:** 2009-09-03

**Authors:** Richard A. Klinghoffer, Jason Frazier, James Annis, Jason D. Berndt, Brian S. Roberts, William T. Arthur, Raul Lacson, Xiaohua Douglas Zhang, Marc Ferrer, Randall T. Moon, Michele A. Cleary

**Affiliations:** 1 Rosetta Inpharmatics, LLC, Seattle, Washington, United States of America; 2 Howard Hughes Medical Institute, Institute for Stem Cell and Regenerative Medicine, and Department of Pharmacology, University of Washington School of Medicine, Seattle, Washington, United States of America; 3 Department of Automated Biotechnology, Merck Research Laboratories, Merck & Co., Inc., North Wales, Pennsylvania, United States of America; 4 Department of Biometrics Research, Merck Research Laboratories, Merck & Co., Inc., West Point, Pennsylvania, United States of America; Charité-Universitätsmedizin Berlin, Germany

## Abstract

The multi-protein β-catenin destruction complex tightly regulates β-catenin protein levels by shuttling β-catenin to the proteasome. Glycogen synthase kinase 3β (GSK3β), a key serine/threonine kinase in the destruction complex, is responsible for several phosphorylation events that mark β-catenin for ubiquitination and subsequent degradation. Because modulation of both β-catenin and GSK3β activity may have important implications for treating disease, a complete understanding of the mechanisms that regulate the β-catenin/GSK3β interaction is warranted. We screened an arrayed lentivirus library expressing small hairpin RNAs (shRNAs) targeting 5,201 human druggable genes for silencing events that activate a β-catenin pathway reporter (BAR) in synergy with 6-bromoindirubin-3′oxime (BIO), a specific inhibitor of GSK3β. Top screen hits included shRNAs targeting dihydrofolate reductase (DHFR), the target of the anti-inflammatory compound methotrexate. Exposure of cells to BIO plus methotrexate resulted in potent synergistic activation of BAR activity, reduction of β-catenin phosphorylation at GSK3-specific sites, and accumulation of nuclear β-catenin. Furthermore, the observed synergy correlated with inhibitory phosphorylation of GSK3β and was neutralized upon inhibition of phosphatidyl inositol 3-kinase (PI3K). Linking these observations to inflammation, we also observed synergistic inhibition of lipopolysaccharide (LPS)-induced production of pro-inflammatory cytokines (TNFα, IL-6, and IL-12), and increased production of the anti-inflammatory cytokine IL-10 in peripheral blood mononuclear cells exposed to GSK3 inhibitors and methotrexate. Our data establish DHFR as a novel modulator of β-catenin and GSK3 signaling and raise several implications for clinical use of combined methotrexate and GSK3 inhibitors as treatment for inflammatory disease.

## Introduction

β-catenin signaling is critical in normal development and adult physiology. Both hyper- and hypoactivation of this pathway have been linked to disease [1], [2]. β-catenin signaling is tightly regulated at the level of intracellular protein accumulation by the β-catenin destruction complex, which consists of glycogen synthase kinase 3β (GSK3β), casein kinase 1 (CK1), the scaffold protein Axin, and the tumor suppressor protein adenomatous polyposis coli (APC) [3]. In the absence of pathway activation by WNT ligands or small molecule inhibitors of GSK3, β-catenin is sequentially phosphorylated by CK1 and GSK3β[4]. Phosphorylated β-catenin is recognized by β-TrCP, a component of the dedicated E3 ubiquitin ligase complex, and following ubiquitination, β-catenin is targeted for rapid degradation by the proteasome [5]. Strong evidence demonstrates that mutations resulting in constitutive activation of β-catenin, promote the initiation and progression of colon cancer. Greater than 80% of colorectal tumors have loss-of-function mutations in APC and many of the remaining tumors harbor gain-of-function mutations in β-catenin itself [6], [7], [8]. While increased β-catenin signaling drives progression of colon cancer, paradoxically, hyper-activation of β-catenin may actually promote survival of melanoma patients by altering melanoma cell fate to a less proliferative state [9], [10]. Several other major human diseases including mood disorders, Alzheimer disease, and osteoporosis may involve reduced β-catenin signaling [1]. In the past several years it has also become clear that GSK3 is a prominent mediator of inflammation, and it is likely that this function of GSK3 underlies the pathology of several disease states [11], [12]. Although much is now known regarding control of β-catenin and GSK3β function, to better understand how to therapeutically modulate this fundamental pathway additional pathway regulators must be identified.

High-throughput RNA interference (RNAi) screening in cultured human cells represents a powerful, unbiased, and comprehensive genetic approach for dissecting the contribution of individual genes to signaling pathways or cellular functions. By use of libraries of chemically synthesized small interfering RNAs (siRNAs), or small hairpin RNAs (shRNAs) expressed from vectors and packaged as pseudotyped viral particles, we and others have shown the utility of RNAi screens to define modulators of clinically relevant and investigative compounds [13], [14], [15]. Because the β-catenin/GSK3 pathway represents a potentially valuable, albeit complex, target of interest, we performed an RNAi screen to identify potentially druggable modulators of a GSK3-specific inhibitor, 6-bromoindirubin-3′oxime (BIO). We screened a library of >13,000 individually arrayed lentiviral shRNA vectors targeting >5,000 druggable human genes for vectors that would activate a β-catenin pathway reporter (BAR) [16] synergistically with a suboptimal dose of BIO. We expect that confirmed hits from this screen may represent novel targets for therapeutically manipulating β-catenin/GSK3 signaling and potentially uncover strategies for combination therapies that may increase the potency of GSK3-specific inhibitors.

## Results

### Screening a lentiviral shRNA library targeting the druggable genome for GSK3 inhibitor enhancers

**Figure 1:**
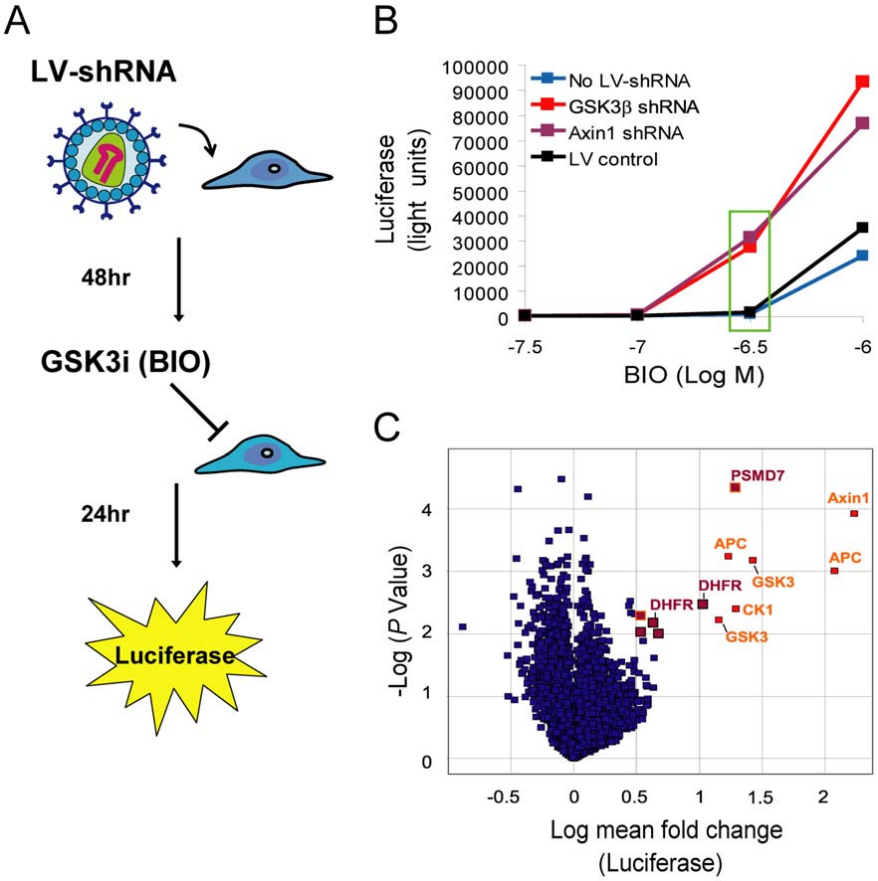
A screen for enhancers of GSK3 inhibitor activity identified shRNAs targeting DHFR among top hits. (A) Schematic of the screening assay using the BAR reporter. (B) Establishment of the optimal dose of BIO for the enhancer screen using RKO-BAR cells. RKO-BAR cells were mock infected (No LV-shRNA), or were infected with lentiviral vectors that expressed no shRNA (LV-control) or shRNAs targeting either GSK3β or Axin1. Forty eight hours after transduction, cells were exposed to a titration of BIO (GSK3i), and luciferase activity was measured after 24 hr of exposure to drug. Silencing of GSK3β or Axin1 enhanced luciferase activity at doses of BIO that showed no detectable luciferase activity in mock and control vector infected cells (green box). This optimized dose range of BIO (300–500 nM) was used in our screen and follow up studies. (C) Volcano plot of the screen using a lentiviral shRNA library targeting the druggable genome. Samples were screened in triplicate in the presence of 312 nM BIO. shRNA vectors that increased luciferase activity by 3-fold or greater with a *P* value less than 0.01 are highlighted as top hits. Among this set of hits, known members of the β-catenin destruction complex (including GSK3β) are colored and labeled in orange, whereas potentially novel GSK3 modulators including the two DHFR shRNAs are colored maroon. The two PSMD7 shRNAs are colored maroon with an orange outline. An expanded hit list with full statistical analysis is provided in Table S1.

We screened RKO colon carcinoma cells that express a β-catenin responsive firefly luciferase reporter (BAR) [16]. Intracellular β-catenin accumulation and subsequent luciferase expression are tightly regulated in this reporter line by an intact β-catenin destruction complex. In these cells, inhibition of this complex by a GSK3-specific inhibitor results in β-catenin accumulation and translocation to the nucleus and robust luciferase expression. We optimized this reporter assay to identify shRNAs that synergize with GSK3 inhibition by establishing a dose of BIO that activates BAR only in concert with a silencing event that impairs GSK3 activity (Figure 1A). To do this we compared BAR activation in cells transduced with an shRNA targeting either GSK3β itself or another gene such as Axin1 that facilitates GSK3 regulation of β-catenin, to untransduced cells or cells transduced with a lentiviral vector control. Although none of the vectors tested resulted in reporter activation in the absence of BIO, cells transduced with shRNA vectors targeting GSK3β or Axin1 consistently enabled a sub-optimal, non-activating dose of BIO (312 nM) to activate BAR luciferase expression compared with vector control and uninfected cells (Figure 1B). We used this concentration of BIO to screen for shRNA enhancers of GSK3 inhibitor activity.

Next we screened an arrayed library of 13,609 lentiviral shRNAs, targeting 5,201 human genes predicted to constitute the druggable genome [17]. Samples were screened in triplicate, and stringent hit calling criteria were set at a normalized mean fold-change of greater than 3 with a Student's t-test *P* value less than 0.01 (Figure 1C, and Table 1). With this data restriction, the screen yielded 12 top hits (0.09% of the library) that resulted in robust reporter activation in concert with a non-activating dose of BIO. Consistent with known biology, these select hits included 6 distinct shRNAs in the library targeting known members of the β-catenin destruction complex (two GSK3β shRNAs, two APC shRNAs, one Axin1 shRNA, and one CK1α shRNA). The remaining top hits were represented by shRNAs targeting potential novel regulators of GSK3 signaling and included two distinct shRNAs targeting dihydrofolate reductase (DHFR), two PSMD7 shRNAs, and single shRNAs targeting ELA2 and RNF10. Reduction of hit-calling criteria stringency (mean fold change >2.5, *P* value <0.05) added third shRNAs targeting GSK3β and DHFR to this list of BIO enhancers (Supplementary Table 1). Because, outside of GSK3β, DHFR was the only target on our hit list represented by three distinct shRNA vectors, and given that DHFR is the target of the widely used anti-inflammatory drug methotrexate [18], we focused hit confirmation and follow up analysis on the potential relationship between DHFR and GSK3.

**Table 1:** Top screen hits for shRNA enhancers of BIO induced BAR activity.

Gene symbol	Functional notes	*P* value	BAR fold change
Axin 1	β-catenin destruction complex	1.2×10-4	172.4
APC	β-catenin destruction complex	9.7×10-4	119.8
GSK3B	β-catenin destruction complex	6.6×10-4	26.6
PSMD7	26S proteasome, putative regulatory subunit	4.4×10-5	19.6
CSNK1A1	β-catenin destruction complex	4.0×10-3	19.4
APC	β-catenin destruction complex	5.7×10-4	16.9
GSK3B	β-catenin destruction complex	6.0×10-3	14.3
DHFR	Folate biosynthesis, target of methotrexate	3.2×10-3	10.7
RNF10	ring finger, transcription factor binding protein	1.0×10-2	4.8
DHFR	Folate biosynthesis, target of methotrexate	6.7×10-3	4.2
ELA2	Elastase 2 neutrophil	9.7×10-3	3.4
PSMD7	26S proteasome, putative regulatory subunit	5.3×10-3	3.4

The gene symbol and functional annotation for each intended shRNA is provided. *P* value and BAR fold change compared with the median luciferase signal of the sample field for each screen plate were calculated as described in experimental procedures.

### DHFR shRNA hit confirmation

**Figure 2:**
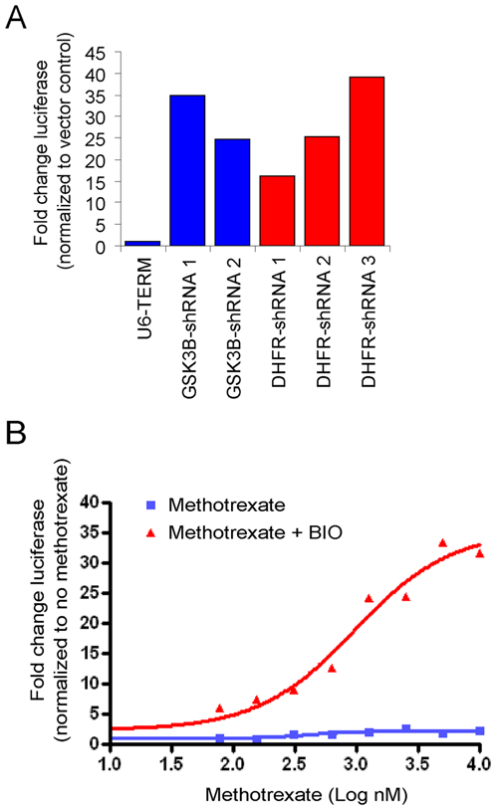
GSK3 inhibitor activity is enhanced by either DHFR silencing or inhibition by methotrexate. (A) Hit confirmation by the BAR assay. Fresh virus generated for DHFR shRNAs, GSK3β shRNAs, and empty vector controls was used to transduce RKO-BAR cells. 48 hours following transduction cells were exposed to a sub-optimal (non-activating) dose of GSK3i (312 nM) for another 24 hours before measuring pathway activation by luciferase activity. Importantly, all three vectors targeting DHFR (red bars) induced similar levels of activation as shRNAs targeting GSK3β. (B) Examination of the effect of the DHFR inhibitor methotrexate on BAR activation by BIO. RKO-BAR cells were exposed to a titration of methotrexate in the presence (red) or absence (blue) of a non-activating dose of BIO and luciferase activity was measured. In confirmation of the enhancement of BIO activity in cells transduced with shRNA targeting DHFR, inhibition by methotrexate is synergistic with GSK3 inhibition for activation BAR.

**Figure 3:**
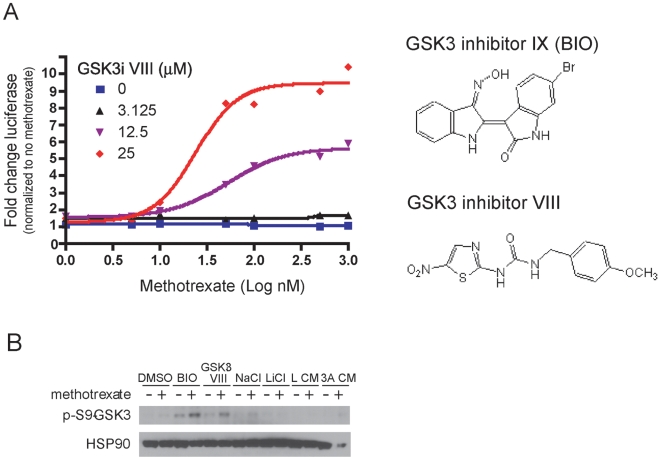
BAR activation induced by dual inhibition of DHFR and GSK3 correlates with increased GSK3β phosphorylation. (A) The experiment was performed as described for BIO in figure legend 2 with the substitution of an alternative GSK3 inhibitor. (B) RKO-BAR cells were exposed to multiple activators of β-catenin signaling (GSK3 specific inhibitors BIO and GSK3i VIII, lithium chloride (LiCL), and Wnt3A conditioned medium (3ACM)) or controls (DMSO, NaCL, and control conditioned medium (LCM)). Protein lysates were harvested and subjected to Western Blot analysis with a GSK3β phospho-serine 9 specific antibody. The enhancement of BAR activity by DHFR inhibition correlated with agents that induced phosphorylation of GSK3 at inhibitory residue serine 9. The result is representative of two separate experiments.

To confirm DHFR as a hit, we regenerated vectors for all three library DHFR shRNA sequences, verified them by DNA sequencing (not shown), and packaged them into lentiviral particles. As expected, in concert with a sub-optimal dose of BIO, the DHFR shRNAs induced robust activation of BAR to levels similar to that induced by shRNAs targeting GSK3β (Figure 2A). The three DHFR shRNA vectors induced target knockdown to 34%, 50%, and 21% (DHFR shRNAs 1, 2, and 3, respectively) of the DHFR mRNA levels in the vector alone (U6-term) control sample. Correlation between phenotype and silencing was reasonable given that the vector that induced the greatest DHFR silencing also resulted in the strongest pathway activation (DHFR shRNA 3). To strengthen our confidence that the observed reporter activation resulted from on-target inhibition of DHFR, we exposed RKO-BAR cells to a titration of methotrexate in the presence or absence of a suboptimal dose of BIO. Neither methotrexate nor the suboptimal dose of BIO alone significantly increased BAR activity. However, when added in combination, reporter activation increased in a manner dependent on increasing concentrations of methotrexate (Figure 2B). Together with the observation that three distinct shRNAs targeting the same gene resulted in the same phenotype, these results support the conclusion that the observed effect is due to on-target inhibition of DHFR. In addition, synergistic BAR activation was also driven by combined exposure of cells to methotrexate and a second structurally unrelated GSK inhibitor (GSK inhibitor VIII, Calbiochem), but not by the combination of methotrexate and lithium or Wnt-3a conditioned medium (Figure 3A). Interestingly the selectivity of the synergistic interaction correlated with increased phosphorylation of an inhibitory residue (Ser-9) on GSK3β induced by BIO or GSK3 inhibitor VIII, but not by lithium or Wnt-3a (Figure 3B). Importantly, while the enhancement may be selective for certain GSK3 inhibitors, the data suggest that the capacity to synergize with DHFR inhibition is not specific to a structural class of GSK3 inhibiting compounds.

### DHFR inhibition enhances accumulation of β-catenin protein by decreasing GSK3-mediated phosphorylation of β-catenin

**Figure 4:**
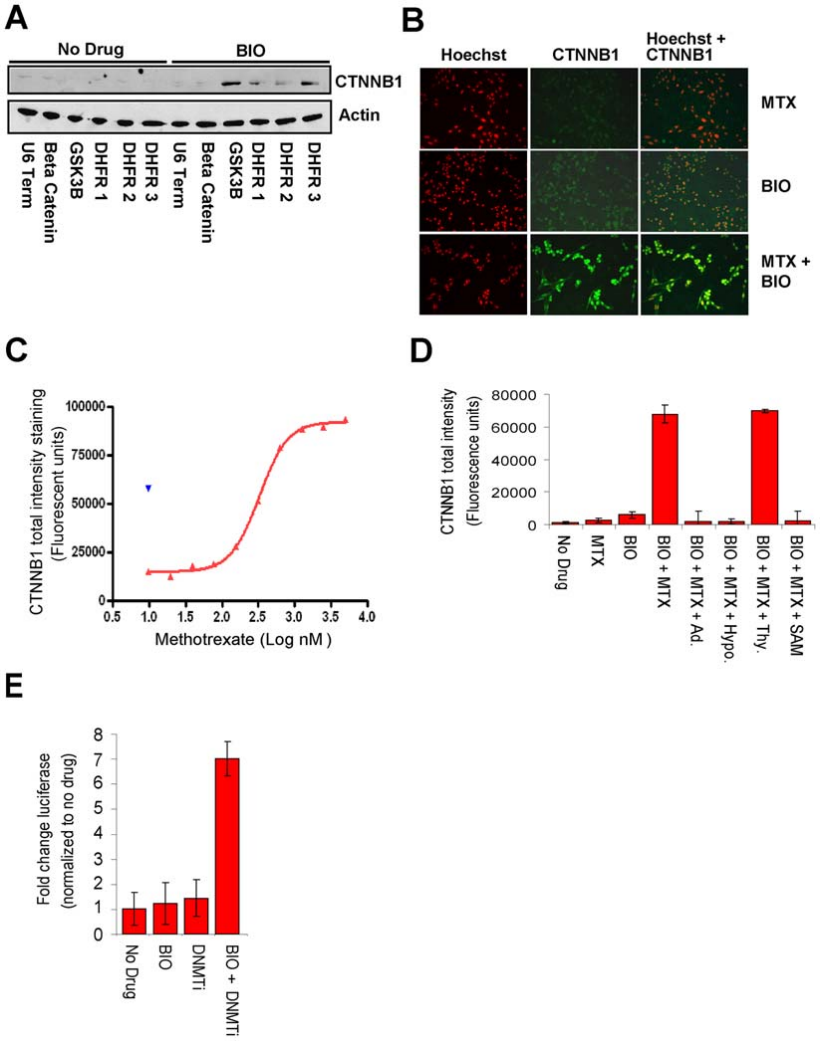
DHFR inhibition synergizes with BIO to increase intracellular β-catenin protein accumulation. (A) RKO-BAR cells were transduced with a lentiviral vector control lacking an shRNA (U6-TERM), with vectors encoding shRNAs targeting β-catenin or GSK3β, or with vectors encoding three distinct shRNAs targeting DHFR and then exposed to BIO (right lanes) or vehicle control (left lanes) 48 hours following transduction. Protein lysates were harvested and subjected to Western Blot analysis with a β-catenin specific antibody. Whereas none of the vectors had an effect in the absence of drug, silencing of GSK3β or DHFR synergized with BIO to induce accumulation of β-catenin (CTNNB1, blot is representative of three separate experiments). (B) RKO-BAR cells were exposed to methotrexate (MTX), BIO (312 nM), or the combination of the two drugs and stained with the nuclear dye Hoechst and with an antibody recognizing β-catenin. Images were collected on an InCELL 1000 high content imager. A synergistic increase in β-catenin expression and nuclear localization was observed upon dual inhibition of GSK3 and DHFR. (C) RKO-BAR cells were exposed to a titration of methotrexate in the presence of 312 nM BIO and stained for β-catenin. β-catenin expression was quantified on an Acumen Explorer laser scanning microplate reader. As shown in the graph, a methotrexate dose-dependent increase in β-catenin levels was observed (red curve, each point represents the average of six replicate samples). The effect of a BAR-activating dose of BIO alone (1 µM) is provided as a reference (blue). (D) RKO-BAR cells were subjected to DHFR, GSK3 (BIO 312 nM), or dual inhibition in the absence or presence of products of the folate metabolism pathway. β-catenin accumulation was analyzed as in panel C. Data represent the mean and standard deviation (SD) of 8 replicate wells for each sample. Addition of purines (adenosine, Ad., or hypoxanthine, Hypo.) or s-adenosylmethionine (SAM), but not thymidine (Thy.) blocked the synergistic effect of dual inhibition. (E) RKO-BAR cells were subjected to DNMT inhibition (DNMTi) with 5′ Aza-2′-deoxycytidine (750 nM), BIO (312 nM), or both inhibitors in combination. Luciferase activity was measured 16 h following drug addition. Data represent the mean±SD of 4 replicate wells for each sample.

To investigate how DHFR inhibition enhanced the effect of GSK3 inhibition, we tested whether inhibition by shRNA silencing or methotrexate led to an increase in β-catenin mRNA and protein levels. To examine these possibilities, we transduced RKO-BAR cells with DHFR shRNA and control viruses, exposed cells to BIO or vehicle control, and then measured the accumulation of β-catenin protein by Western blot and β-catenin mRNA by Q-PCR. Consistent with an impact on the β-catenin destruction complex, silencing of DHFR increased β-catenin protein levels in concert with BIO, but no increase in the transcript was observed (Figure 4A and Figure S1). This result was confirmed further by the dose-dependent nuclear accumulation of β-catenin following exposure of cells to a sub-optimal concentration of BIO and a titration of methotrexate measured by immunofluorescent staining with a β-catenin specific antibody and the nuclear dye Hoechst (Figure 4B and 4C).

DHFR regulates the regeneration of tetrahydrofolate, a carrier of one-carbon units used in a variety of biosynthetic processes including synthesis of pyrimidines, S-adenosylmethionine (SAM), and purines [19]. Addition of adenosine, hypoxanthine, and SAM to cells exposed to methotrexate and GSK3 inhibitor completely ablated β-catenin accumulation, whereas addition of thymidine had no effect (Figure 4D). Furthermore, three shRNAs targeting thymidylate synthetase within our screening library failed to enhance GSK3-inhibitor driven BAR activation. These observations are consistent with a role for DHFR in regulating GSK3 activity via synthesis of purines and/or SAM, but not pyrimidines and suggest that the impact of DHFR inhibition on GSK3 is unlikely to result from inhibition of DNA synthesis or repair, both of which require intact thymidine synthesis. Consistent with the possibility that the enhancement of BIO by DHFR inhibition is mediated, at least in part, by perturbation of DNA methylation, co exposure of cells to BIO and the DNMT inhibitor 5′-Aza-2′-deoxycytidine resulted in modest (7-fold) but synergistic activation of BAR (Figure 4E).

Given that addition of SAM blocked methotrexate enhancement of BIO-induced β-catenin protein accumulation, we questioned whether aberrant methylation may impact expression of GSK3β or other members of the destruction complex. Measurement of mRNA levels of Axin1, Axin2, β-TrPC, CK1α, CUL4b, and GSK3β by Q-PCR revealed no substantial changes following shRNA- or methotrexate-induced inhibition of DHFR alone or in combination with BIO compared to controls (Figure S1). Although we cannot rule out the possibility that DHFR inhibitor-induced changes in mRNA expression have some impact on GSK3 regulation, our data strongly indicate that changes in the transcript levels of GSK3 or other prominent destruction complex members do not underlie the observed synergy with BIO.

**Figure 5:**
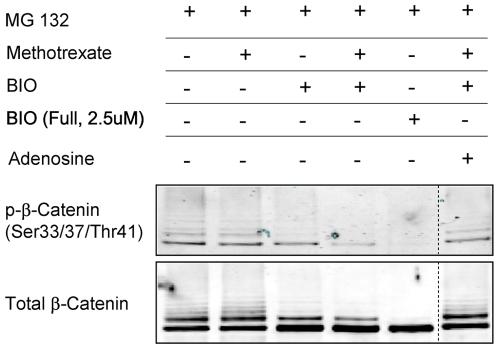
Methotrexate enhances BIO-mediated inhibition of phosphorylation at GSK3-specific residues (Ser33/37/Thr41) on β-catenin. RKO-BAR cells were treated with the proteasome inhibitor MG132 alone (1 µM), or in combination with other compounds as depicted, and subjected to immunoprecipitation with a β-catenin specific antibody followed by Western blot for total and phospho- (Ser33/37/Thr41) β-catenin. Exposure of cells to suboptimal BIO (312 nM) plus methotrexate (312 nM) led to a greater decrease in phosphorylated β-catenin than exposure to the same dose of BIO alone (compare lanes 4 and 5). As observed with β-catenin accumulation (Figure 2), further exposure to adenosine (100 µM) neutralized the impact of methotrexate on BIO-mediated inhibition of β-catenin phosphorylation. The dashed line denotes that the adenosine treated sample is from a separate part of the same blot. This result is representative of three separate experiments.

We next examined whether inhibition of DHFR could interfere with the ability of GSK3 to phosphorylate substrates such as β-catenin. Because GSK3 regulates β-catenin stability by phosphorylating β-catenin serine and threonine residues (Ser33/37 and Thr41) we examined whether DHFR inhibition affected this phosphorylation event. To enable a comparison of the phosphorylation status of β-catenin between cells with active GSK3 versus potentially inhibited GSK3, β-catenin degradation was blocked in all samples by treatment with the proteasome inhibitor MG132. In line with our previous experiments, exposure to methotrexate had no affect on β-catenin protein levels or phosphorylation status compared with control cells treated with MG132 alone. In contrast, treatment of cells with a fully active concentration of BIO (2.5 µM) completely inhibited GSK3-mediated phosphorylation of β-catenin (Ser33/37/Thr41) as well as laddering of total β-catenin on the protein gel, which is suggestive of decreased β-catenin ubiquitination. Importantly, cells treated with the dose of BIO used in our screen (312 nM) exhibited only modest decreases in β-catenin phosphorylation and laddering, and these effects were significantly enhanced by co-treatment with methotrexate (Figure 5). Similar to our previous experiments, the enhancement of BIO by methotrexate was neutralized by addition of adenosine. Together, these data indicate that DHFR inhibition and subsequent inhibition of the purine and/or SAM synthesis arms of the folate metabolism pathway lead to impaired GSK3 activity and decreased phosphorylation of β-catenin.

**Figure 6:**
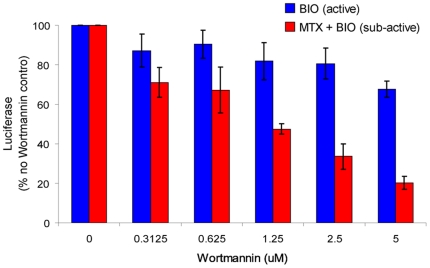
Methotrexate enhancement of BIO-mediated BAR activation is blocked by the PI3K inhibitor Wortmannin. RKO-BAR cells treated with either suboptimal BIO (312 nM) plus methotrexate (red bars), or with an active concentration of BIO (1.5 µM, blue bars), were exposed to a titration of Wortmannin, and luciferase activity was measured 24 h following exposure to drug. Consistent with a requirement for PI3K signaling to mediate the synergistic interaction between DHFR and GSK3 inhibitors, wortmannin significantly reduced BAR activity induced by the combination of the two inhibitors in a dose dependent manner. In contrast wortmannin had modest effect on BAR activity induced by the active dose of BIO alone. Results represent the mean±SD of three replicate wells for each sample.

Phosphorylation and inactivation of GSK3β occur in response to growth factor stimulation of receptor tyrosine kinases and subsequent activation of PI3K signaling. Since enhancement of GSK3 inhibitor-mediated BAR activation by methotrexate correlated with increased inhibitory phosphorylation of GSK3β (Figure 3), we examined whether the observed synergy required PI3K activity. Consistent with a requirement for PI3K signaling to mediate the interaction between DHFR and GSK3 inhibitors, exposure of cells to the PI3K inhibitor wortmannin blocked BAR activation induced upon coexposure to methotrexate and BIO (Figure 6, red bars). A similar response was observed upon exposure of cells to a second PI3K inhibitor, LY294002 (not shown). Interestingly, the impact of PI3K inhibition was specific to BAR activation induced by dual inhibition of DHFR and GSK3, as wortmannin has little impact on BAR activation induced by an active concentration of BIO (Figure 6, blue bars). Preliminary gene expression profiling experiments from our laboratory suggest that methotrexate-induced expression of multiple growth factors and growth factor receptors could drive activation of PI3K. However the exact mechanism regarding the interplay between DHFR, GSK3, and PI3K requires further investigation.

### Methotrexate enhances the anti-inflammatory effect of GSK3 inhibition

**Figure 7:**
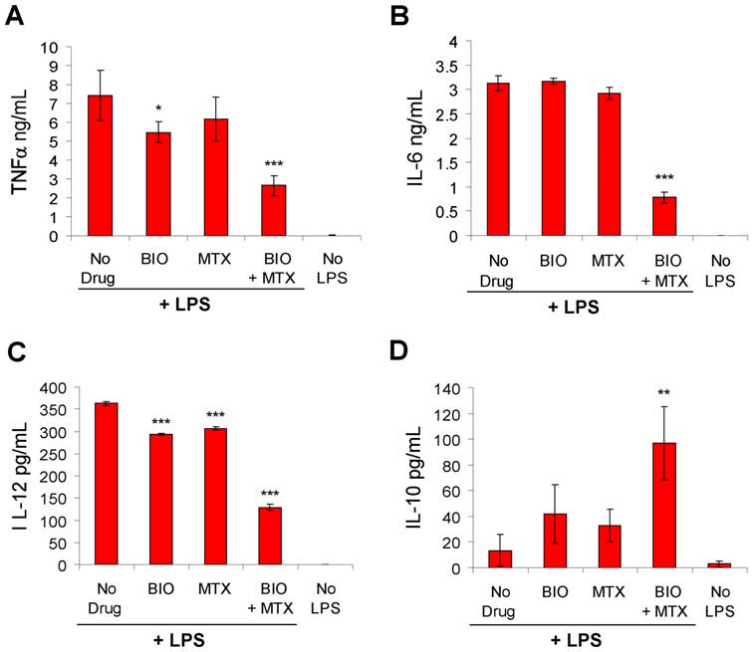
DHFR inhibition enhances pro and anti-inflammatory cytokine production by inhibition of GSK3. Cytokine production in PBMCs preincubated for 1 hr in medium only (No Drug), 5 nM BIO (GSK3i), 10 nM methotrexate (MTX), or the combination of the two compounds (BIO + MTX) and then stimulated with 300 ng/ml LPS for 20 hr. No LPS controls are shown on the far right of each graph for reference. Cell free supernatants were analyzed by ELISA for production of TNFα (A), IL-6 (B), IL-12 (C), or IL-10 (D). *, *P*<0.05, **, *P*<0.01, and ***, *P*<0.001, compared with LPS alone. Results represent the mean±SD of 6 replicate wells for each sample. The results shown are representative of three separate experiments.

GSK3 promotes pro-inflammatory signaling downstream of Toll-like receptor (TLR) activation, and GSK3-specific inhibitors have been shown to significantly decrease TLR-mediated inflammatory responses. Pre-treatment of primary immune cells with GSK3 inhibitors substantially increases production of the anti-inflammatory cytokine IL-10 while suppressing production of pro-inflammatory cytokines including TNFα, IL-6, and IL-12 [11]. Because methotrexate is widely used to treat inflammation-related disorders such as rheumatoid arthritis, and our data show that DHFR inhibition increases the potency of GSK3 inhibitors, we tested whether methotrexate could enhance the anti-inflammatory effects of GSK3 inhibition on peripheral blood mononuclear cells (PBMCs). To investigate potential synergy, we first assessed the anti-inflammatory effect of each drug as a single agent. PBMCs were pre-treated with a titration of either BIO or methotrexate, prior to TLR activation by LPS stimulation, and drug concentrations resulting in sub-maximal modulation of LPS-induced cytokine production were chosen for assessment of synergy (data not shown). Consistent with the previously observed capacity of methotrexate to enhance the potency of BIO in RKO cells, LPS-induced production of pro-inflammatory cytokines was severely blunted, whereas production of IL-10 was significantly increased in PBMCs pre-treated with the drug combination compared with cells pre-treated with either methotrexate or BIO alone (Figure 7). Importantly the effect of dual inhibition exceeded the additive effect of each drug as a single agent indicating that dual inhibition of DHFR and GSK3 results in a synergistic anti-inflammatory effect.

## Discussion

High-throughput RNAi screening provides an unbiased and powerful means to interrogate attractive drug targets with complex signaling functions. Such screens can reveal unexpected interactions that impact mechanisms of target regulation and can ultimately promote advancement strategies for target-specific inhibitors in the clinic. A growing body of literature indicates that positive modulation of β-catenin signaling has potential in the treatment of a wide range of diseases, and small molecule inhibitors of GSK3 represent one means to potentiate this pathway [1]. Moreover, recent evidence has focused on GSK3 as a potential target for treating inflammation-related conditions [11], [12]. Our screen revealed the intriguing observation of synergy between a GSK3 specific inhibitor, BIO, and shRNA molecules that silence DHFR, the target of the widely used anti-inflammatory drug methotrexate. This finding highlights a novel interaction between folate metabolism and β-catenin/GSK3 signal transduction pathways and suggests potential opportunities for the use of DHFR and GSK3 inhibitors in the clinic.

To our knowledge, this is the first report of modulation of GSK3 activity by inhibition of DHFR. However, links between folic acid intake and β-catenin have been observed previously. Patients with rectal adenomas treated with dietary folic acid supplementation for one year exhibited reduced nuclear translocation of β-catenin and reduced cellular expression of inactive phospho-Ser9-GSK3β compared with placebo treated controls [20]. Also intriguing is the finding that the Crooked tail (Cd) model of human folate-responsive neural tube defects is mutated in Wnt coreceptor Lrp6 [21]. Furthermore, significantly increased levels of β-catenin protein and nuclear localization were observed upon examination of the colonic mucosa of mice subjected to combined depletion of folate and B-vitamins for a period of 10 weeks [22]. This effect was attributed to DNA hypomethylation and subsequent induction of strand breaks within the APC gene leading to decreased expression of APC mRNA. Consistent with this observation, mice expressing hypomorphic alleles of DNMT1 that result in reduced genomic methylation, exhibit an increased incidence of APC LOH event and increase β-catenin accumulation in microadenomas of the colon [23]. Although we did not observe an obvious effect on APC expression upon inhibition of DHFR, the results described in the two aforementioned studies are consistent with our observation that cellular exposure to SAM, the major donor of one-carbon units in cellular methylation reactions, blocked the synergistic accumulation of β-catenin that occurred in response to dual inhibition of DHFR and GSK3. The lack of effect on APC expression in our studies may be due to the short duration of our assays (hours – days) compared with the longer studies performed in the mouse models and in the clinic (weeks – 1 year). Our data suggest that early consequences of decreased folate metabolism, mediated at least in part by decreased cellular methylation, can lead to a more immediate impact on β-catenin regulation by GSK3 than indicated previously.

The mechanism of the interaction between DHFR inhibition and β-catenin/GSK3 is likely to be complex and due to cross-talk between these signaling pathways rather than direct activation of GSK3 by DHFR. Although DHFR inhibition increased the potency of BIO, in the absence of BIO, methotrexate and RNAi-induced silencing of DHFR had no effect BAR activity, β-catenin accumulation, or β-catenin phosphorylation. Evidence shown here suggests that alterations in methylation caused by DHFR inhibition may underlie the synergy with BIO. However, to what extent the observed synergy is due to changes in DNA methylation versus changes in protein methylation, or both, remains to be determined. The observation that exposure to adenosine blocked the synergistic effect of combined DHFR and GSK3 inhibition opens the possibility that signaling events outside of those perturbed by changes in methylation may play a role in regulating pathway cross-talk. To date, four key mechanisms have been identified that contribute to regulation of GSK3. These include regulation by phosphorylation of GSK3 itself, the subcellular localization of GSK3, protein-protein interactions with GSK3, and the phosphorylation status of GSK3 substrates [24]. The data presented here suggest the possibility that inhibition of folate metabolism primes cells for GSK3β inhibition and subsequent β-catenin activation by upregulating activators of PI3K signaling.

Given the recent demonstration of anti-inflammatory properties of GSK3 inhibitors, and the use of low dose methotrexate as the gold standard treatment for rheumatoid arthritis, our data raise several interesting possibilities for the use of these inhibitors in the clinic. The observation that dual inhibition of GSK3 and DHFR led to a synergistic anti-inflammatory response suggests that the combination of DHFR and GSK3 inhibitors may have merit for use in the clinic. However, it is unclear whether the potent anti-inflammatory effect of combined inhibition requires activation of β-catenin signaling. The ability to separate the anti-inflammatory and pro-oncogenic effects of dual DHFR and GSK3 inhibition will ultimately determine the utility of the proposed combination therapy for treatment of chronic inflammatory conditions. Interestingly, rheumatoid arthritis patients treated with methotrexate were found to have a 50% increased risk of developing certain cancers [25]. Increased β-catenin signaling provides a plausible molecular mechanism for this increased risk in patients on methotrexate. While increased β-catenin signaling has clearly been linked to development of certain cancers such as colon cancer, two studies in malignant melanoma suggest that elevated nuclear β-catenin may improve survival [9], [10]. It is possible that depending on the cellular context, β-catenin can promote either oncogenic proliferation or de-differentiation of tumor cells into a less pathologic state. In the latter case, agents such as methotrexate that synergize with β-catenin pathway activators may have a therapeutic effect in the oncology clinic. Further mechanistic understanding of the interactions between DHFR, GSK3, and β-catenin should lead to insights as to how to best modulate GSK3 as a drug target.

## Materials and Methods

### Reagents

293FT producer cells (Invitrogen, Carlsbad, CA) were maintained as per the manufacturer's specifications in DMEM supplemented with 10% FBS, and 0.1 mM MEM non-essential amino acids, 2 mM L-glutamine, 1% Pen/strep, and 500 µg/ml Geneticin (Invitrogen). HT29 (ATCC, Rockville, MD) and RKO-BAR cells engineered to express a β-catenin responsive firefly luciferase reporter (BAR)[16] were maintained in DMEM supplemented with 10% fetal bovine serum. Primary human PBMCs (Astarte Biologics, Redmond, WA) were cultured in RPMI supplemented with 10% FBS. Several chemical compounds and antibodies were used in this study: BIO and GSK inhibitor VIII (Calbiochem, La Jolla, CA); methotrexate, adenosine, hypoxanthine, thymidine, s-adenosylmethionine, and total β-catenin antibody (Sigma Aldrich, St. Louis, MO); phospho-β-catenin (Ser33/37/Thr41) antibody and phospho-GSK3β (Ser-9) antibody (Cell Signaling Technology). The shRNAs consisting of a 19mer stem with a 9-base hairpin loop were cloned into the pLentiLox3.7 vector and were designed using standard asymmetry rules [26]. Virus packaging was achieved with ViraPower™ plasmid mix (Invitrogen). The following DHFR shRNA sequences were used in this study: DHFR1 (5′- GTAGACATGGTCTGGATAG-3′), DHFR2 (5′-GACTTTGAAAGTGACACGT-3′), and DHFR3 (5′- GGTAAACAGAATCTGGTGA-3′).

### Virus production and assay for viral titer

Automated high-throughput lentiviral vector production was performed as described previously [15]. 13, 609 individual shRNA vectors targeting 5, 201 human genes (typical coverage of 3 shRNAs per gene) were packaged as lentivirus particles. The pLentilox3.7 vector encodes EGFP, which was used to assess virus production for each library sample following infection of HT29 colon carcinoma cells, our standard line for establishing lentivirus titer (Figures S2 and S3).

### High-throughput lentivirus-mediated RNAi screen

Infection conditions were optimized for screening in 384-well format. Robot-assisted lentiviral infection of RKO-BAR cells was carried out as follows. One set of 180 96-well infection-ready plates (IRPs) comprising the screening library was thawed on ice and kept at 4°C prior to infection. 384-well screening plates were primed for infection by addition of 15 µl of plating medium (DMEM, 10% FBS, 25 mM HEPES, Pen/Strep) to each well with a WellMate® bulk dispensing apparatus (Matrix Technologies, Hudson, NH). Next, four IRPs were then transferred (10 µl of virus/well) into each screening plate in triplicate by use of a Biomek® FX Laboratory Automation Workstation (Beckman Coulter, Fullerton, CA). RKO-BAR cells were harvested by trypsinization and reverse transduced by seeding of the virus-containing screening plates at a density of 1200 cells/well in the presence of 6 µg/ml polybrene in a final volume of 35 µl of growth medium using the bulk dispenser. Forty-eight hours after infection, BIO was added to a final concentration of 312 nM and firefly luciferase was measured by use of a BriteLite luciferase assay (PerkinElmer, Waltham, MA) 24 hours later.

### Statistical analysis

Smoothing methods were used to adjust for positional effects including systematic edge effects [27], [28]. In each plate, the difference between the adjusted intensity of an shRNA and the median in the sample field was calculated [29]. For all shRNA replicates, these values were used to calculate mean difference (equivalent to mean fold change in log scale), strictly standardized mean difference (SSMD) and *P* value of testing no mean difference [30], [31], [32]. The *P* values were then used to calculate the false discovery rate (FDR) by use of methods described by Strimmer [33].

### Quantitative PCR

mRNA silencing was quantified by real-time PCR with an ABI PRISM 7900HT Sequence Detection System and Assays-on-Demand™ gene expression products as described previously [13]. The following Assays-on-Demand™ reagents were used in this study: DHFR (Hs00758822_s1), GSK3β (Hs00275656_m1), β-catenin (Hs00170025_m1), Axin1 (Hs00394718_m1), APC (Hs00181051_m1), Axin2 (Hs00610344), βTrPC (Hs00182707_m1), Cul4B (Hs00186086_m1), CK1α (Hs00793391_m1).

#### Measurement of cytokines

Human IL-6, IL-10, and TNFα in culture supernatants were measured with enzyme-linked immunosorbent assay (ELISA) kits from Invitrogen. IL-12 was measured with an ELISA kit from Pierce, Rockford, IL.

## Supporting Information

Figure S1 DHFR inhibition does not decrease mRNA expression of known components of the β-catenin destruction complex. RKO-BAR cells were transduced with DHFR shRNAs or were exposed to methotrexate with or without BIO and RNA was harvested for Q-PCR analysis with probes specific for Axin1, Axin2, β-TRPC, CK1α, CUL4b, and GSK3β. Of the genes tested, a modest, albeit consistent, reduction in Axin2 expression alone was observed upon silencing and methotrexate inhibition of DHFR. However, as expected with activation of β-catenin, exposure to the combination of BIO and methotrexate led to an increase in Axin2 expression, and Axin2 shRNAs did not lead to GSK3 inhibitor enhancement in our screen. It is therefore unlikely that a reduction in Axin2 levels alone underlies the synergistic interaction between DHFR and GSK3 inhibitors.
(0.04 MB DOC)

Figure S2 Establishment of minimal titer values in GFP units required to detect enhancement of BIO-mediated activation of BAR by GSK3β shRNA. Because it is difficult to normalize viral titers across a large collection of packaged vectors we established a minimal titer threshold that would be expected to result in BAR activation if gene silencing enhanced BIO potency. To establish a minimum effective titer, we exposed HT29 cells to serially diluted control virus (U6-TERM, which contains the U6 promoter and two tandem RNA-polymerase III termination sequences but no shRNA) or virus targeting GSK3β. GFP expression was measured 96 h post transduction to determine titer values in terms of fluorescent light units on an Acumen Explorer laser scanning microplate reader (TTP LabTech, Cambridge U.K.). In parallel with the HT29 infections, RKO-BAR cells were infected with same serially diluted control virus or lentiviral shRNA targeting GSK3β. Forty eight hours after infection, cells were exposed to BIO at a concentration (312 nM) that induces luciferase-based luminescence only upon infection with an shRNA capable of negatively modulating GSK3 function. Significance of enhanced activation of luciferase by the GSK3β shRNA infected cells over that observed in control vector infected cells is plotted on the Y-axis. GFP values as Log total intensity fluorescent units as a measurement of viral titer is plotted on the X-axis. Each data point represents the comparison of 4 replicate wells for each dilution of U6-TERM to GSK3β shRNA vector. As expected, we observed a strong correlation between titer of GSK3β shRNA virus and enhancement of luciferase activation by BIO. The arrow denotes the titer value in GFP units (1.7×107 FLU) required for 5-fold enhancement of luciferase activation with a P value of 1×10-4. We set this as our minimal titer value for effective lentiviral shRNA production for this screen.
(0.03 MB DOC)

Figure S3 Analysis of titer values for the lentiviral shRNA screening library. Titer values were established based on GFP expression as described in Figure S2. On the basis of the minimal effective titer values established (described in Figure S1, red line), all but 77 of our library wells (99.5%) met levels required to enable enhancement of BIO in our assay assuming efficacy of shRNA-induced target silencing and impact of GSK3 function. Each green dot represents an individually produced and analyzed virus. The entire library was analyzed by use of this method. The bottom panel is an enlargement of the section encompassed by the blue box in the top graph.
(0.07 MB DOC)

Table S1 Statistical analysis of top shRNA screen hits. Gene Symbol and ID numbers for the intended targets of each shRNA screen hit are provided. Hits are ranked by strictly standardized mean difference (SSMD) using a cut-off of SSMD >1.6. All statistical metrics were calculated as described in procedures. Each hit listed represents a distinct shRNA sequence and the three DHFR shRNA vectors are highlighted in bold.
(0.03 MB DOC)
