# Supplementary material for: A Lentivirus-Mediated Genetic Screen Identifies Dihydrofolate Reductase (DHFR) as a Modulator of β-Catenin/GSK3 Signaling
Source: PLoS One. 2009 Sep 3;4(9):e6892. doi: 10.1371/journal.pone.0006892 (PMC2731218; doi:10.1371/journal.pone.0006892)
Supplement: Figure S1 — DHFR inhibition does not decrease mRNA expression of known components of the β-catenin destruction complex. RKO-BAR cells were transduced with DHFR shRNAs or were exposed to methotrexate with or without BIO and RNA was harvested for Q-PCR analysis with probes specific for Axin1, Axin2, β-TRPC, CK1α, CUL4b, and GSK3β. Of the genes tested, a modest, albeit consistent, reduction in Axin2 expression alone was observed upon silencing and methotrexate inhibition of DHFR. However, as expected with activation of β-catenin, exposure to the combination of BIO and methotrexate led to an increase in Axin2 expression, and Axin2 shRNAs did not lead to GSK3 inhibitor enhancement in our screen. It is therefore unlikely that a reduction in Axin2 levels alone underlies the synergistic interaction between DHFR and GSK3 inhibitors. (0.04 MB DOC) [file pone.0006892.s001.doc]

**Figure S1.** **DHFR inhibition does not decrease mRNA expression of known components of the β-catenin destruction complex.** RKO-BAR cells were transduced with DHFR shRNAs or were exposed to methotrexate with or without BIO and RNA was harvested for Q-PCR analysis with probes specific for Axin1, Axin2, β-TRPC, CK1α, CUL4b, and GSK3β. Of the genes tested, a modest, albeit consistent, reduction in Axin2 expression alone was observed upon silencing and methotrexate inhibition of DHFR. However, as expected with activation of β-catenin, exposure to the combination of BIO and methotrexate led to an increase in Axin2 expression, and Axin2 shRNAs did not lead to GSK3 inhibitor enhancement in our screen. It is therefore unlikely that a reduction in Axin2 levels alone underlies the synergistic interaction between DHFR and GSK3 inhibitors.
