# Supplementary material for: A Lentivirus-Mediated Genetic Screen Identifies Dihydrofolate Reductase (DHFR) as a Modulator of β-Catenin/GSK3 Signaling
Source: PLoS One. 2009 Sep 3;4(9):e6892. doi: 10.1371/journal.pone.0006892 (PMC2731218; doi:10.1371/journal.pone.0006892)
Supplement: Figure S2 — Establishment of minimal titer values in GFP units required to detect enhancement of BIO-mediated activation of BAR by GSK3β shRNA. Because it is difficult to normalize viral titers across a large collection of packaged vectors we established a minimal titer threshold that would be expected to result in BAR activation if gene silencing enhanced BIO potency. To establish a minimum effective titer, we exposed HT29 cells to serially diluted control virus (U6-TERM, which contains the U6 promoter and two tandem RNA-polymerase III termination sequences but no shRNA) or virus targeting GSK3β. GFP expression was measured 96 h post transduction to determine titer values in terms of fluorescent light units on an Acumen Explorer laser scanning microplate reader (TTP LabTech, Cambridge U.K.). In parallel with the HT29 infections, RKO-BAR cells were infected with same serially diluted control virus or lentiviral shRNA targeting GSK3β. Forty eight hours after infection, cells were exposed to BIO at a concentration (312 nM) that induces luciferase-based luminescence only upon infection with an shRNA capable of negatively modulating GSK3 function. Significance of enhanced activation of luciferase by the GSK3β shRNA infected cells over that observed in control vector infected cells is plotted on the Y-axis. GFP values as Log total intensity fluorescent units as a measurement of viral titer is plotted on the X-axis. Each data point represents the comparison of 4 replicate wells for each dilution of U6-TERM to GSK3β shRNA vector. As expected, we observed a strong correlation between titer of GSK3β shRNA virus and enhancement of luciferase activation by BIO. The arrow denotes the titer value in GFP units (1.7×10^7^ FLU) required for 5-fold enhancement of luciferase activation with a P value of 1×10^-4^. We set this as our minimal titer value for effective lentiviral shRNA production for this screen. (0.03 MB DOC) [file pone.0006892.s002.doc]

**Figure S2.** **Establishment of minimal titer values in GFP units required to detect enhancement of BIO-mediated activation of BAR by GSK3β shRNA.**  Because it is difficult to normalize viral titers across a large collection of packaged vectors we established a minimal titer threshold that would be expected to result in BAR activation if gene silencing enhanced BIO potency. To establish a minimum effective titer, we exposed HT29 cells to serially diluted control virus (U6-TERM, which contains the U6 promoter and two tandem RNA-polymerase III termination sequences but no shRNA) or virus targeting GSK3β.  GFP expression was measured 96 h post transduction to determine titer values in terms of fluorescent light units on an Acumen Explorer laser scanning microplate reader (TTP LabTech, Cambridge U.K.). In parallel with the HT29 infections, RKO-BAR cells were infected with same serially diluted control virus or lentiviral shRNA targeting GSK3β. Forty eight hours after infection, cells were exposed to BIO at a concentration (312 nM) that induces luciferase-based luminescence only upon infection with an shRNA capable of negatively modulating GSK3 function. Significance of enhanced activation of luciferase by the GSK3β shRNA infected cells over that observed in control vector infected cells is plotted on the Y-axis. GFP values as Log total intensity fluorescent units as a measurement of viral titer is plotted on the X-axis. Each data point represents the comparison of 4 replicate wells for each dilution of U6-TERM to GSK3β shRNA vector. As expected, we observed a strong correlation between titer of GSK3β shRNA virus and enhancement of luciferase activation by BIO. The arrow denotes the titer value in GFP units (1.7 x 10^7^ FLU) required for 5-fold enhancement of luciferase activation with a *P* value of 1 x 10^-4^. We set this as our minimal titer value for effective lentiviral shRNA production for this screen
