# Supplementary material for: A Lentivirus-Mediated Genetic Screen Identifies Dihydrofolate Reductase (DHFR) as a Modulator of β-Catenin/GSK3 Signaling
Source: PLoS One. 2009 Sep 3;4(9):e6892. doi: 10.1371/journal.pone.0006892 (PMC2731218; doi:10.1371/journal.pone.0006892)
Supplement: Figure S3 — Analysis of titer values for the lentiviral shRNA screening library. Titer values were established based on GFP expression as described in Figure S2. On the basis of the minimal effective titer values established (described in Figure S1, red line), all but 77 of our library wells (99.5%) met levels required to enable enhancement of BIO in our assay assuming efficacy of shRNA-induced target silencing and impact of GSK3 function. Each green dot represents an individually produced and analyzed virus. The entire library was analyzed by use of this method. The bottom panel is an enlargement of the section encompassed by the blue box in the top graph. (0.07 MB DOC) [file pone.0006892.s003.doc]

**Figure S3.** **Analysis of titer values for the lentiviral shRNA screening library.** Titer values were established based on GFP expression as described in Figure S1. On the basis of the minimal effective titer values established (described in Figure S2, red line), all but 77 of our library wells (99.5 %) met levels required to enable enhancement of BIO in our assay assuming efficacy of shRNA-induced target silencing and impact of GSK3 function. Each green dot represents an individually produced and analyzed virus. The entire library was analyzed by use of this method. The bottom panel is an enlargement of the section encompassed by the blue box in the top graph.
