# Supplementary material for: A Lentivirus-Mediated Genetic Screen Identifies Dihydrofolate Reductase (DHFR) as a Modulator of β-Catenin/GSK3 Signaling
Source: PLoS One. 2009 Sep 3;4(9):e6892. doi: 10.1371/journal.pone.0006892 (PMC2731218; doi:10.1371/journal.pone.0006892)
Supplement: Table S1 — Statistical analysis of top shRNA screen hits. Gene Symbol and ID numbers for the intended targets of each shRNA screen hit are provided. Hits are ranked by strictly standardized mean difference (SSMD) using a cut-off of SSMD >1.6. All statistical metrics were calculated as described in procedures. Each hit listed represents a distinct shRNA sequence and the three DHFR shRNA vectors are highlighted in bold. (0.03 MB DOC) [file pone.0006892.s004.doc]

**Table S1. Statistical analysis of top shRNA screen hits.** Gene Symbol and ID numbers for the intended targets of each shRNA screen hit are provided. Hits are ranked by strictly standardized mean difference (SSMD) using a cut-off of SSMD > 1.6. All statistical metrics were calculated as described in procedures. Each hit listed represents a distinct shRNA sequence.
